# Connective tissue disease-associated interstitial lung disease: immune profiling and correlation of immune cells with pulmonary function in SSc-ILD

**DOI:** 10.1080/07853890.2026.2745489

**Published:** 2026-10-15

**Authors:** Zhenkun Yang, Huixing Li, Chu Qin, Yuanyuan Luo, Li Zhu, Ying Yin, Jingyu Chen, Zhening Pu, Tingting Wang

**Affiliations:** ^a^ Department of Laboratory Medicine, The Affiliated Wuxi People’s Hospital of Nanjing Medical University, Wuxi, Jiangsu, China; ^b^ Wuxi Medical Center, Nanjing Medical University, Wuxi, Jiangsu, China; ^c^ Department of Thoracic Surgery, The Affiliated Wuxi People’s Hospital of Nanjing Medical University, Wuxi, Jiangsu, China; ^d^ Department of Respiratory, The Affiliated Wuxi People’s Hospital of Nanjing Medical University, Wuxi, Jiangsu, China

**Keywords:** Connective tissue diseases, Interstitial lung disease, Immune, Pulmonary function, Flow cytometry

## Abstract

**Background:**

Connective tissue diseases (CTDs) frequently involve the lungs, with CTD-associated interstitial lung disease (CTD-ILD) representing a leading cause of morbidity and mortality, yet the immunopathological mechanisms remain incompletely defined. This study characterized immune profiles in CTD-ILD and their association with pulmonary function impairment, focusing on systemic sclerosis-associated ILD (SSc-ILD).

**Methods:**

This single-center retrospective study enrolled 217 CTD-ILD patients, 100 healthy controls, and 25 idiopathic pulmonary fibrosis (IPF) patients. Peripheral lymphocyte subsets, immunoglobulins, complements, and cytokines were assessed using flow cytometry and immunoturbidimetry. Lung function was evaluated by standard spirometry and gas transfer tests.

**Results:**

Compared to healthy controls, lymphocyte counts were reduced in all ILD subtypes, lowest in polymyositis/dermatomyositis (PM/DM)-ILD. In SSc-ILD, T cell and helper T cell (Th) percentages were significantly elevated (76.2% and 49.39%, respectively). Complement C3 was lower in Sjögren’s syndrome (SS)-ILD (889.92 vs. 965.49 mg/L), and C4 was significantly lower in SSc-ILD (196.74 vs. 226.70 mg/L) versus controls. SSc-ILD patients with higher Th cell proportions exhibited worse pulmonary function, with expiratory reserve volume (ERV) falling 57.21%, maximal vital capacity (VC max) 18.81%, functional residual capacity (FRC) 19.64%, forced vital capacity (FVC)17.93%, and diffusing capacity for carbon monoxide (DLCO) 20.28%. Continuous correlation analysis further confirmed significant negative correlations between Th cell proportions and ERV, FRC, and PCO_2_.

**Conclusion:**

Peripheral blood immune profiling revealed distinct abnormalities across CTD-ILD subtypes. Peripheral Th cell expansion is strongly associated with lung function decline in SSc-ILD, highlighting the potential of Th cells as biomarkers of disease progression in SSc-ILD.

## Introduction

Connective Tissue Diseases (CTDs), including systemic lupus erythematosus (SLE), rheumatoid arthritis (RA), Sjögren’s syndrome (SS), systemic sclerosis (SSc), and polymyositis/dermatomyositis (PM/DM), comprise a heterogeneous group of systemic autoimmune disorders that affect approximately 10% of the population, with a marked female predominance [1]. CTDs are characterized by dysregulated immune responses, wherein autoantibodies and aberrantly activated immune cells contribute to chronic inflammation and progressive tissue damage across multiple organ systems [2]. Complex interactions between the innate and adaptive immune systems promote the release of pro-inflammatory cytokines and profibrotic mediators, ultimately leading to endothelial injury, tissue fibrosis, and organ dysfunction [3]. Therefore, analyzing immunity is crucial for understanding the mechanism and progression of CTDs.

Among the various organs affected, the lungs are commonly involved and represent a major determinant of prognosis. CTD-associated interstitial lung disease (CTD-ILD) is a significant cause of morbidity and mortality, occurring in 10–20% of patients with RA, 9–24% with SS, and up to 70% with PM/DM and SSc [4]. Histopathologically, CTD-ILD is characterized by alveolar inflammation, epithelial-mesenchymal transition, and progressive fibrosis, driven by immune cell infiltration and dysregulated tissue repair mechanisms [5]. As previously described, patients with CTD-ILD have an accumulation of T and B lymphocytes in the lung tissue and bronchoalveolar lavage [6,7]. As key orchestrators of adaptive immunity, T lymphocytes regulate immune responses through direct cell-cell interactions and cytokine secretion, facilitating fibroblast activation, extracellular matrix deposition, and fibrotic remodeling [8]. Notably, cytokines secreted by T cells, such as interleukin-13 (IL-13), promote fibroblast differentiation into myofibroblasts, thereby accelerating fibrosis [9]. Immune dysregulation is the primary pathological factor in the progression of CTD-ILD; in addition to T cells, various immune cells, including B cells and macrophages, also play a role in its occurrence and development [10]. Recent advancements in high-throughput transcriptomics have uncovered some distinct immune cell signatures in CTD-ILD. For instance, autoreactive B cells are believed to contribute to a profibrotic microenvironment in SSc-ILD, while impaired clearance of apoptotic cells and dysregulated extracellular matrix (ECM) turnover by macrophages may also drive disease progression [11,12]. Given the immunologically complex nature of CTD-ILD, in-depth profiling of immune cell composition and function is essential to better understand disease mechanisms.

In this retrospective single-center study, we integrated clinical and laboratory data from CTD-ILD patients to systematically investigate peripheral and pulmonary immune profiles and their relationship with lung function. Our objective was to identify immune correlates of disease severity and bridge the gap between molecular mechanisms and clinical outcomes in CTD-ILD.

## Methods

### Patient population and enrollment

This retrospective, single-center study reviewed medical records of patients diagnosed with both CTD and ILD at the Affiliated Wuxi People’s Hospital of Nanjing Medical University between January 2020 and May 2025. The data analysis was conducted between September 2025 and January 2026. Overall, a total of 217 CTD-ILD patients were enrolled, including 95 with PM/DM-ILD, 58 with SS-ILD, 35 with RA-ILD, and 29 with SSc-ILD. All patients met internationally recognized classification criteria for CTDs and had radiologically confirmed ILD based on high-resolution computed tomography (HRCT) findings. Exclusion criteria included the presence of other autoimmune or chronic inflammatory diseases (e.g. asthma, psoriasis, type 1 diabetes), non-ILD pulmonary manifestations (e.g. pulmonary arterial hypertension), malignancy, organ transplantation, or incomplete clinical/laboratory data. Additionally, 100 healthy individuals with no known pulmonary or systemic autoimmune disease were recruited as controls, and 25 patients with idiopathic pulmonary fibrosis (IPF) were recruited for further investigation. This retrospective study has received approval from the Ethics Committee of the Affiliated Wuxi People’s Hospital of Nanjing Medical University (Approval No. KY26054) in accordance with China’s Good Clinical Practice guidelines and the Declaration of Helsinki. All study subjects were de-identified in advance to remove any personally identifiable information, while patients retained the right to withdraw their consent at any time; this opt-out methodology waived the requirement for written informed consent.

### Laboratory assessments and pulmonary function testing

The peripheral blood immune characteristics were detected during the first visit of patients diagnosed with CTD-ILD at our hospital. Serum levels of immunoglobulins (IgA, IgG, IgM) and complements (C3, C4) were measured by immunoturbidimetry on an automated biochemical detection workstation (Beckman Coulter, CA, USA).

Cytokine concentrations (IL-2, IL-4, IL-6, IL-10, IFN-γ, TNF-α, IL-17) were assessed *via* flow cytometry using a Th cytokine panel (Cell-genebio, Hangzhou, China). Briefly, 25 µL of capture beads, 25 µL of fluorescent detection reagent, and 25 µL of serum sample were sequentially added to the bottom of a flow cytometry tube. The mixture was gently vortexed and incubated at room temperature in the dark for 2.5 h. After incubation, 1 mL of phosphate-buffered saline (PBS) was added to each tube, followed by centrifugation at 200 × g for 5 min. The supernatant was carefully discarded, and the bead pellet was resuspended in 100 µL PBS. Data acquisition was performed on a flow cytometer (NAVIOS; Beckman Coulter, CA, USA). Commercial quality control (QC) samples at two concentration levels (low and high) were run daily before sample analysis.

In addition, the pulmonary function tests were performed concurrently with peripheral blood sampling during the same clinical visit, with an interval of no more than one week, to evaluate the correlation between immune function and pulmonary function.

### Lymphocyte subset analysis

T, B, and natural killer (NK) cell subsets were quantified using a 6-color TBNK Reagent Kit (Tongsheng Shidai, Beijing, China) according to the manufacturer’s protocol. The following fluorochrome-conjugated monoclonal antibodies (mouse-derived) and clones were used: CD3-FITC (UCHT1), CD16+CD56-PE (CB16, MEM-188), CD45-PerCP-Cy5.5 (2D1), CD4-PC7 (RPA-T4), CD8-APC-Cy7 (HIT8a), and CD19-APC (HIB19). All antibodies were purchased from BD Biosciences (CA, USA). Briefly, 20 µL of monoclonal antibody cocktail was added to the bottom of a flow cytometry tube. Using the reverse pipetting technique, 50 µL of EDTA-K2 anticoagulated whole blood was gently mixed and then transferred to the tube. The mixture was gently vortexed and incubated at room temperature in the dark for 15 min. Subsequently, 450 µL of lysis buffer was added, gently vortexed, and incubated at room temperature in the dark for another 15 min. Data acquisition was performed on a flow cytometer (FACSCanto II; BD, NJ, USA), and analysis was carried out using Kaluza software (Beckman Coulter). A total of 5,000 lymphocytes were collected for each sample. Daily quality control was performed using commercial QC beads to verify instrument performance, including laser alignment, fluorescence intensity, and signal stability; commercial QC blood samples at two concentration levels (low and high) were run daily before sample analysis. Coefficients of variation for all lymphocyte subset assays were maintained below 4% for markers with mean values exceeding 30%, and below 8% for markers with mean values below 30%, throughout the study period.

### Statistical analysis

Quantitative results are presented as mean ± standard deviation (SD). Comparisons between two groups were performed using Student’s *t*-test, and comparisons among multiple groups were analyzed using one-way analysis of variance (ANOVA) followed by the Least Significant Difference (LSD) post hoc test. Prior to analysis, normality of the data was verified using the Shapiro-Wilk test. Pearson correlation was used to assess the linear relationships. For variables that did not meet the normality assumption, Spearman’s rank correlation was applied instead. Statistical significance was set at *p < 0.05, **p < 0.01. Statistical analyses were conducted using SPSS version 20.0 (IBM, NY, USA), and data visualization was performed using GraphPad Prism version 8.0 (GraphPad Software, CA, USA).

## Results

### Clinical characteristics and laboratory test results of patients with CTD-ILD

Among the 217 CTD-ILD patients enrolled, including PM/DM-ILD, SS-ILD, RA-ILD, and SSc-ILD, 69.12% were female, consistent with the known sex distribution of CTDs [13]. The mean age at diagnosis was 62.89 years, with RA-ILD patients being the oldest on average, and this difference was statistically significant compared to SSc-ILD patients (67.09 vs. 59.59 years, *p* < 0.05). Furthermore, the time from CTD onset to ILD also differed significantly among the subtypes, with PM/DM patients having a mean interval of 1,380 days, significantly shorter than that of other CTD patients (Table 1). Additional clinical information, including smoking history and prior treatment, was also presented, but no statistically significant differences were observed among the four groups.

**Table 1.** Characteristics of all patients.

Characteristic		PM/DM-ILD	SS-ILD	RA-ILD	SSc-ILD	*p* value
Gender	Male (30.88%)	34 (35.79%)	12 (20.69%)	13 (37.14%)	8 (27.59%)	0.196
	Female (69.12%)	61 (64.21%)	46 (79.31%)	22 (62.86%)	21 (72.41%)	
Age (years)		62.01 ± 11.18	63.60 ± 10.57	67.09 ± 9.81^1^	59.59 ± 12.28	0.036
Disease duration (days, N)		1380 ± 2024 (80)	2242 ± 2103 (48)^2^	4049 ± 4356 (34)^3^	4015 ± 3132 (28)^4^	< 0.001
Smoking history (N, Total)		5 (78)	2 (47)	4 (34)	4 (28)	0.294
Prior treatments (N, Total)	UT	7 (79)	8 (48)	3 (34)	2 (27)	0.069
	HT	15 (79)	12 (48)	1 (34)	3 (27)	
	HT+IT	57 (79)	28 (48)	30 (34)	22 (27)	

UT, Untreated; HT, Hormonotherapy; IT, Immunosuppressant therapy. RA-ILD vs SSc-ILD: ^1^*p* < 0.05, PM/DM-ILD vs. SS-ILD: ^2^*p* < 0.05, PM/DM-ILD vs. RA-ILD: ^3^*p* < 0.001, PM/DM-ILD vs. SSc-ILD: ^4^*p* < 0.001.

Autoantibodies are a characteristic feature of CTD and are typically highly disease-specific. In the present study, autoantibody testing revealed that antinuclear antibodies (ANA) were highly prevalent across CTD-ILD subtypes, particularly in SSc-ILD (96.3%) and SS-ILD (92.2%). Disease-specific autoantibodies were also observed: Sjögren’s-associated antibody A/B (SS-A/SS-B) in SS-ILD (positive in 78.18% and 25.45%, respectively), anti-scleroderma 70 antibody (Scl-70) in SSc-ILD (39.29%), and anti-Jo-1 antibody (Jo-1) in PM/DM-ILD (10.47%) (Table 2).

**Table 2.** The positive rate of autoantibodies in each group.

Characteristic	PM/DM-ILD	SS-ILD	RA-ILD	SSc-ILD
ANA (Pos, %, Total)	70.73 (82)	92.16 (51)	66.67 (30)	96.30 (27)
u1-SnRNP (Pos, %, Total)	3.49 (86)	5.45 (55)	10 (30)	14.29 (28)
SS-A (Pos, %, Total)	46.51 (86)	78.18 (55)	16.67 (30)	35.71 (28)
SS-B (Pos, %, Total)	4.65 (86)	25.45 (55)	0 (30)	3.57 (28)
Scl-70 (Pos, %, Total)	0 (86)	1.82 (55)	0 (30)	39.29 (28)
CENP-B (Pos, %, Total)	3.49 (86)	10.91 (55)	13.33 (30)	14.29 (28)
Jo-1 (Pos, %, Total)	10.47 (86)	0 (55)	3.33 (30)	0 (28)

ANA, anti-nuclear antibody; u1-SnRNP, anti-u1 small nuclear ribonucleoprotein complexes antibody; SS-A, Sjögren’s associated antibody A; SS-B, Sjögren’s associated antibody B; Scl-70, anti-scleroderma 70 antibody; CENP-B, anti-centromere protein B antibody; Jo-1, anti-Jo-1 antibody.

CTDs are a group of systemic diseases, and numerous factors in plasma can reflect the body’s stress state. Compared with the normal reference range, inflammatory markers showed elevation of erythrocyte sedimentation rate (ESR) and C-reactive protein (CRP) in all subgroups (Table 3). RA-ILD patients exhibited the highest CRP and lowest albumin (ALB) levels, indicating a more severe inflammatory status compared to other CTD-ILD phenotypes.

**Table 3.** Laboratory examination of all patients.

Characteristic	PM/DM-ILD	SS-ILD	RA-ILD	SSc-ILD	*p* value
ESR (mm/h)	35.94 ± 25.37	36.04 ± 28.83	44.60 ± 30.17	34.82 ± 18.74	0.352
CRP (mg/L)	20.04 ± 29.77	9.56 ± 19.52	25.66 ± 33.16	17.70 ± 28.71	0.047
ALB (g/L)	34.02 ± 5.22^1^	36.40 ± 4.17^2^	31.45 ± 4.71^3^	35.25 ± 5.36	< 0.001

ESR, erythrocyte sedimentation rate, reference range: male 0–15 mm/h, female 0–20 mm/h; CRP, C-reactive protein, reference range: < 0.5 mg/L; ALB, albumin, reference range: 35–50 g/L. PM/DM-ILD vs SS-ILD: ^1^*p* < 0.05; SS-ILD vs RA-ILD: ^2^*p* < 0.0001; RA-ILD vs SSc-ILD: ^3^*p* < 0.05.

### Lymphocyte analysis of the peripheral blood in CTD-ILD and IPF

Abnormalities in the proportions and counts of peripheral blood immune cells (especially T cells, B cells, and their subsets) directly reflect systemic immune dysregulation and are closely associated with the progression of fibrosis [14]. The above clinical data indicated that disease duration differed significantly among the four CTD-ILD subtypes. Therefore, we first compared the peripheral blood lymphocyte proportions among the four CTD-ILD subtypes after adjusting for disease duration. The results showed that the proportions of T cells (CD45^+^CD3^+^) were highest in the SSc-ILD group compared with PM/DM-ILD and SS-ILD. Notably, helper T cells (Th, CD45^+^CD3^+^CD4^+^CD8^-^) were highest in the SSc-ILD group (mean proportion: 49.15%), while the proportion of cytotoxic T cells (Tc, CD45^+^CD3^+^CD4^-^CD8^+^) was highest in the RA-ILD group (mean proportion: 31.62%) compared with other CTD-ILDs (Supplementary Figure 1A). Furthermore, results indicated that the PM/DM-ILD patients had a higher proportion of NK cells (CD45^+^CD3^-^CD16+CD56^+^) compared with SSc-ILD patients, whereas no statistically significant differences were observed for B cell (CD45^+^CD3^-^CD19^+^) proportions or lymphocyte counts (Supplementary Figure 1B, C). These findings suggested that T cells, particularly Th cells and Tc cells, may play important roles in the progression of SSc-ILD and RA-ILD, respectively.

As IPF is an indispensable category among ILDs, this study explored the immune characteristics of peripheral blood in patients with CTD-ILD and IPF. First, the mean lymphocyte count in the normal healthy population was 1.959 × 10^9^/L, and the counts were significantly reduced in all CTD-ILD subtypes compared to healthy individuals, particularly in PM/DM-ILD, SS-ILD, and RA-ILD, with the lowest value observed in the PM/DM-ILD group (mean lymphocyte count: 1.381 × 10^9^/L) (Figure 1C). In SSc-ILD, the percentages of T cells were significantly elevated compared with other disease groups and healthy controls excluding RA-ILD, with mean proportions of 76.2% and 49.39%, respectively. Notably, Th cells were significantly elevated in SSc-ILD compared with other CTD-ILDs and healthy controls, whereas IPF patients (mean proportion: 32.76%) exhibited a significantly decreased level relative to other CTD-ILDs and healthy controls. Regarding the proportion of Tc, the RA-ILD group exhibited the highest percentage among CTD-ILDs and healthy population, with a mean value of 30.64%, but no statistically significant difference compared with IPF patients (Figure 1A). With respect to humoral and innate compartments, compared to the healthy population, B cell percentages were elevated only in IPF, with a mean proportion of 17.2%, and were relatively lower in RA-ILD and SSc-ILD compared to PM/DM-ILD, SS-ILD, and IPF. NK cells showed no significant difference across ILDs and healthy controls, but SSc-ILD exhibited slightly reduced NK cell levels compared to PM/DM-ILD and IPF (Figure 1B).

**Figure 1.**
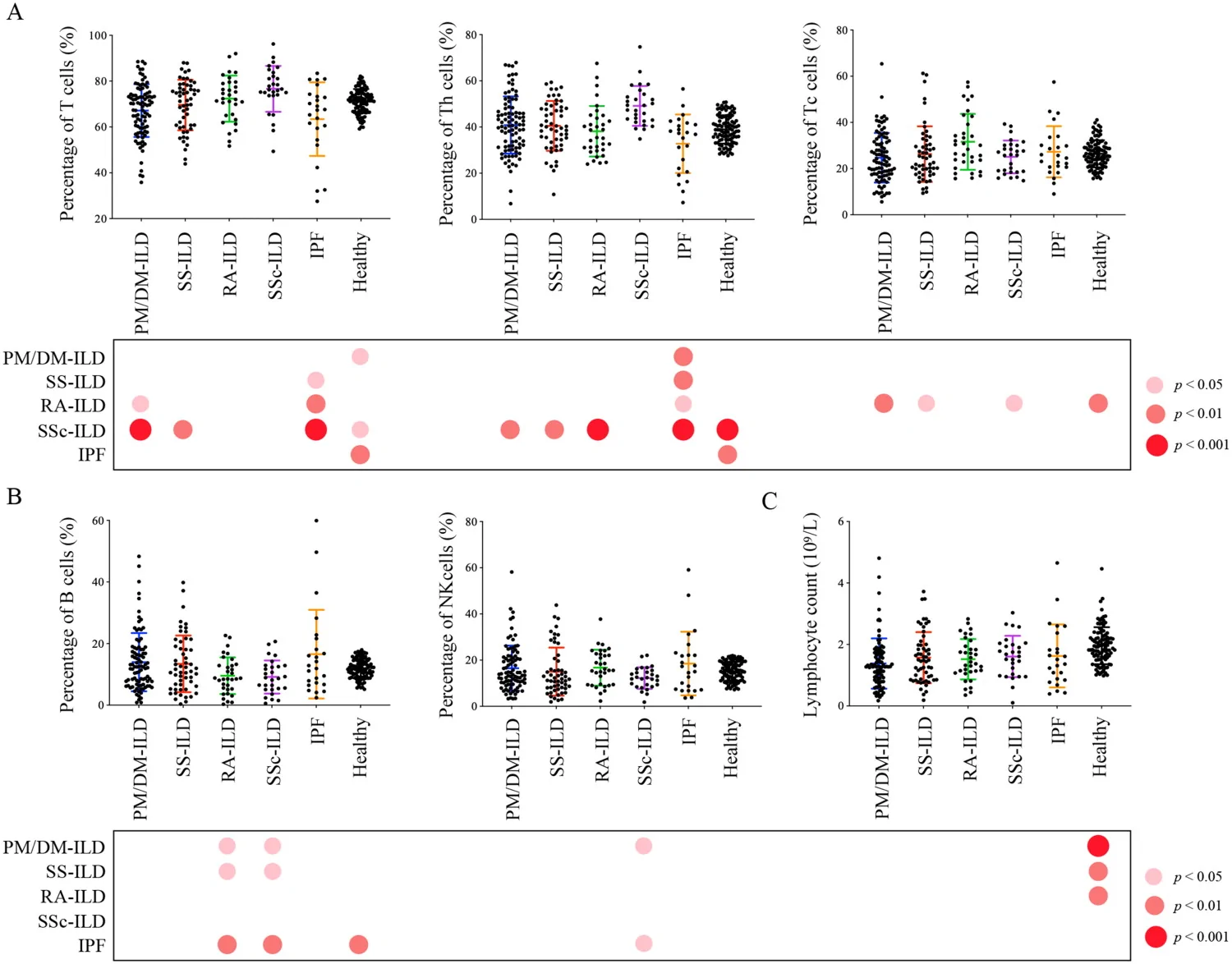
Percentages of circulating immune cells in patients with CTD-ILD and IPF. A. Proportions of T cells, T helper (Th) cells, and cytotoxic T (Tc) cells in peripheral blood. B. Percentages of B cells and natural killer (NK) cells across disease groups. C. Lymphocyte counts in the peripheral blood of ILD patients and healthy controls.

### Cytokines and complements analysis of the peripheral blood in CTD-ILD and IPF

Cytokines and complements act as critical soluble mediators linking cellular immunity to tissue inflammation and fibrosis. We therefore analyzed their circulating levels across all study groups. Immunoglobulin analysis revealed that IgG showed the most pronounced variation among the groups. IgG levels were significantly elevated in SS-ILD and IPF patients compared with healthy controls, with a mean value of 16.11 g/L and 16.65 g/L; whereas RA-ILD patients exhibited the lowest IgG levels among all ILD subtypes and healthy controls (significant differences compared with SS-ILD, SSc-ILD, and IPF), with a mean value of 12.08 g/L. SS is a systemic autoimmune disease characterized by lymphocytic infiltration of exocrine glands. In this study, SS-ILD and RA-ILD patients exhibited higher levels of IgA compared to PM/DM-ILD and healthy individuals. The comparisons of other immunoglobulins and the statistical analysis results among the groups are presented in Figure 2A.

**Figure 2.**
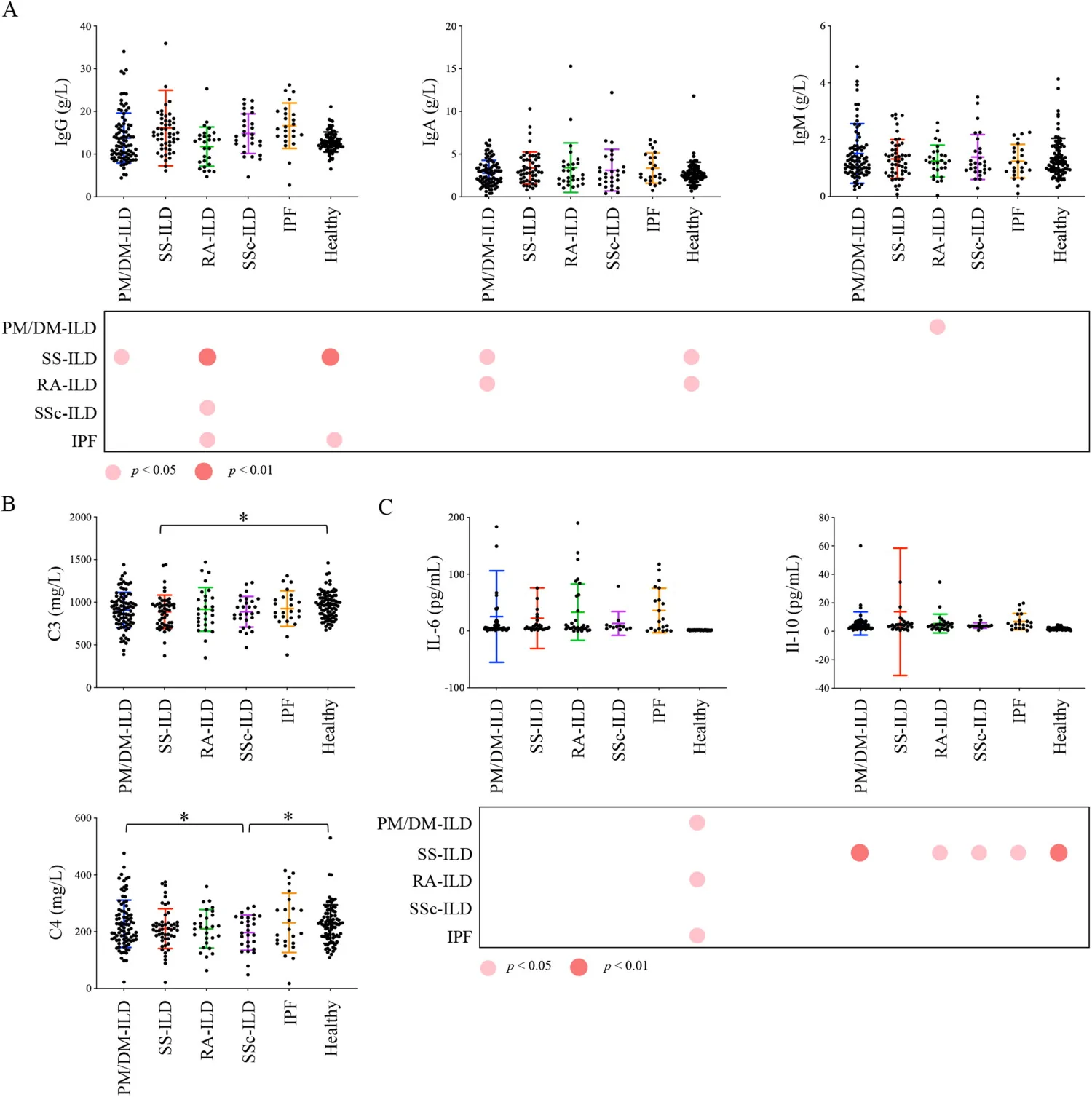
Levels of immunoglobulins, cytokines, and complements in patients with CTD-ILD and IPF. A. Serum levels of immunoglobulins (IgA, IgG, IgM) in the indicated groups. B. Concentrations of complement proteins C3 and C4 in patient plasma. *p < 0.05. C. Cytokine concentrations (IL-6, IL-10) in plasma samples.

Complement is an important mediator of autoimmune diseases. However, the results of this study showed that among all CTD-ILDs, peripheral blood C3 levels were lower than those in healthy controls and were significantly reduced in SS-ILD patients (889.92 vs. 965.49 mg/L); and peripheral blood C4 levels in SSc-ILD patients were significantly lower than those in PM/DM-ILD and healthy controls (Figure 2B). Cytokine network imbalance is a core component of immunopathology and can reflect disease activity. In this study, cytokine profiling revealed that IL-4 levels were higher in all CTD-ILD subgroups except RA-ILD compared to healthy controls. IL-2 and TNF-α were significantly elevated only in the RA-ILD patients (2.96 vs. 1.03 pg/mL and 4.26 vs. 1.01 pg/mL, respectively), while IFN-γ was higher in SS-ILD patients compared to healthy controls (20.36 vs. 0.78 pg/mL) (Supplementary Figure 2). In addition, IL-6 levels were significantly elevated in the PM/DM-ILD, RA-ILD, and IPF groups compared to healthy controls. Meanwhile, IL-10 was significantly higher in the SS-ILD group compared to other CTD-ILDs, IPF, and healthy controls, with a mean value of 19.07 pg/mL (Figure 2C). These data collectively demonstrate that CTD-ILD and IPF share immune dysregulation but possess distinct immunologic features.

### Correlation between immune cells and pulmonary function in SSc-ILD

Given the observed increase in Th cells in SSc-ILD, we further investigated their clinical relevance. Stratification based on peripheral Th cell percentages (the median was used as the cutoff value) revealed that patients with higher Th levels exhibited significantly worse spirometric performance, including reduced expiratory reserve volume (ERV), maximal vital capacity (VC max), and functional residual capacity (FRC). Respectively, ERV decreased by 57.21%, VC max decreased by 18.81%, and FRC decreased by 19.64% (Figure 3A). Similarly, forced vital capacity (FVC) and diffusing capacity for carbon monoxide (DLCO) were significantly decreased in patients with high Th cell proportions, with FVC decreased by 17.93% and DLCO decreased by 20.28% (Figure 3B), suggesting a strong negative correlation between Th cell frequency and lung function. Results of other pulmonary function tests, such as tidal volume (VT) and inspiratory capacity (IC), were shown in Supplementary Figure 3A; however, none of these parameters showed statistically significant differences. Furthermore, arterial blood gas analysis showed reduced partial pressure of carbon dioxide (PCO_2_) in these patients, indicating potential hyperventilation; however, there were no statistically significant differences in partial pressure of oxygen (PO_2_) and oxygen saturation of hemoglobin (SO_2_) between the two groups (Figure 3C, Supplementary Figure 3B). Further continuous correlation analyses revealed that Th cell proportions were significantly negatively correlated with ERV, FRC, and PCO_2_, with correlation coefficients meeting the threshold of |*r*| > 0.3 and *p* < 0.05 (Figure 3D). These findings suggest that elevated peripheral Th cells are associated with impaired pulmonary function and may serve as a biomarker for disease severity in SSc-ILD.

**Figure 3.**
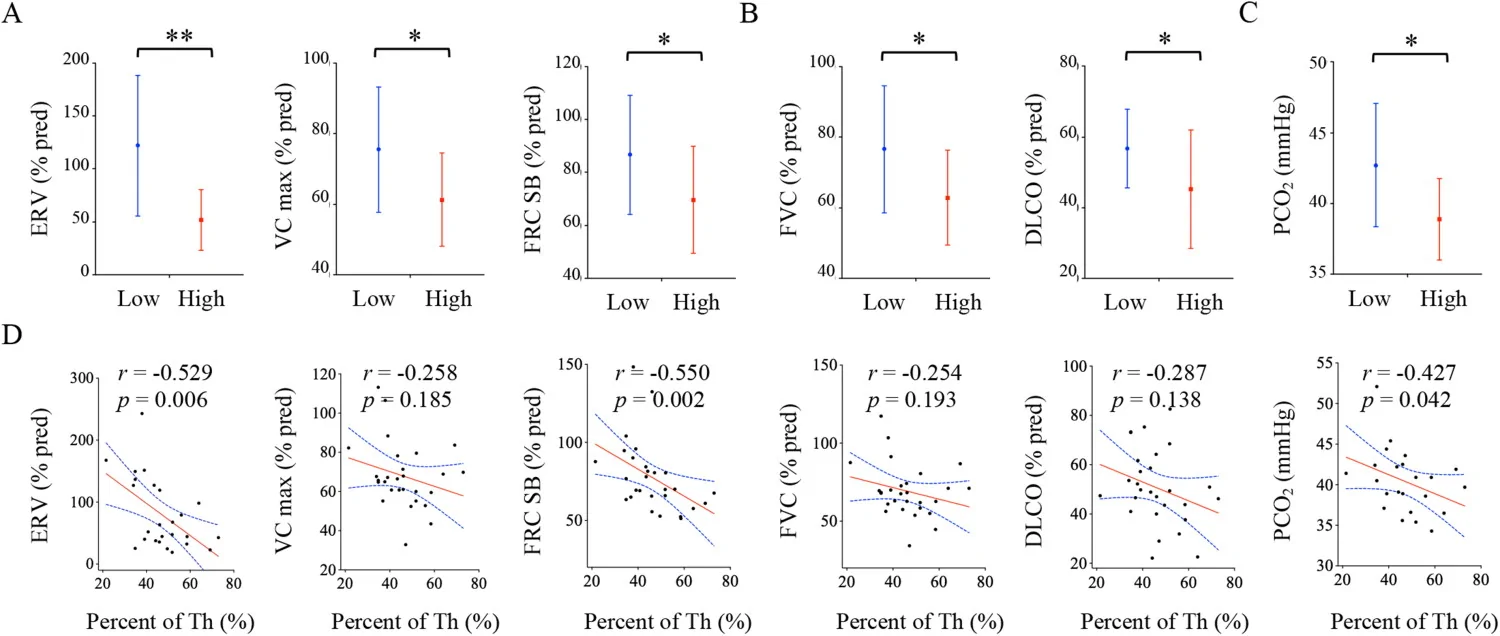
Correlation of Th cells with lung function in SSc-ILD. A. Associations between Th cell proportions and spirometric parameters: expiratory reserve volume (ERV), maximal vital capacity (VC max), and functional residual capacity (FRC). *p < 0.05, **p < 0.01. B. Associations between Th cell proportions and lung volume: forced vital capacity (FVC) and diffusing capacity for carbon monoxide (DLCO). *p < 0.05. C. Relationship between Th cell levels and arterial partial pressure of carbon dioxide (PCO_2_). *p < 0.05. D. Continuous correlation analysis between peripheral blood Th cell proportions and pulmonary function in SSc-ILD patients.

Furthermore, we investigated the clinical relevance of other immune cells. Results revealed that patients with higher Tc levels exhibited significantly reduced maximum voluntary ventilation (MVV), with a decrease of 26.78% (Supplementary Figure 4A); among B cells, both split-by-median analysis and continuous correlation analyses showed that B cells were positively correlated with ERV and FRC, with ERV and FRC increasing by 110.86% and 29.83%, respectively (Supplementary Figure 4B); our results also showed that NK cell proportion was positively correlated with FRC, and the high NK cell proportion group exhibited elevated PCO_2_ levels (Supplementary Figure 4C). This suggests that immune cells are closely associated with the progression of pulmonary interstitial disease and influence lung function.

## Discussion

Immune-mediated processes represent the hallmark of CTDs. In the lungs, immune-driven alveolar and microvascular injury promotes the activation and expansion of resident and recruited immune cells, triggering profibrotic cascades that result in aberrant tissue remodeling and fibrosis [15]. In this study, we provided a peripheral immunological characterization of CTD-ILD, with an emphasis on SSc-ILD, and demonstrated a strong association between peripheral immune alterations and pulmonary function decline. Additionally, to further explore the pulmonary immune microenvironment, we also performed transcriptomic analysis of lung tissues from SSc-ILD, RA-ILD, and IPF patients using publicly available datasets (Supplementary Figure 5). Notably, we identified Th cells as key immune correlates of disease severity in SSc-ILD, with elevated Th cell percentages significantly associated with reduced lung volumes and impaired gas-exchange capacity. This study elucidates the immunopathological characteristics of different CTD-ILDs and suggests that routine monitoring of peripheral blood Th cell proportions may help identify SSc-ILD patients at high risk of progressive pulmonary function deterioration, thereby guiding timely intervention.

Autoantibodies are a hallmark of CTDs; beyond aiding diagnosis, they are associated with clinically significant disease complications, including internal organ involvement [16]. In this study, our analysis identified consistent enrichment of immunoglobulin-related pathways in RA-ILD, SSc-ILD, and IPF (Supplementary Figure 5C), and we further observed that peripheral blood immunoglobulin levels were significantly elevated in CTD-ILD and IPF patients compared with healthy individuals. Although IPF is not classically defined as an autoimmune condition, elevated immunoglobulin levels and autoantibodies have been reported and may correlate with disease progression [17,18]. In the present study, both transcriptomic data (Supplementary Figure 5D) and peripheral blood analysis confirmed increased B cell activity in IPF. Previous histopathological studies demonstrated prominent B cell infiltration and tertiary lymphoid structure formation in IPF lungs, implicating B cells in fibrogenesis [19], providing new insights into IPF therapy.

In CTDs, autoantibodies not only serve as diagnostic biomarkers but are also mechanistically linked to tissue injury and disease severity [16]. For instance, elevated levels of anti-cyclic citrullinated peptide antibody (ACPA) in RA-ILD have been associated with more aggressive pulmonary involvement, and were identified as the strongest predictor of RA-ILD [20,21]. B cells and plasma cells were also increased in the pulmonary interstitium in RA-ILD. In addition, our findings indicated that Th2, Tc central memory, and mast cells were present in higher numbers in the pulmonary interstitium of IPF and RA-ILD (Supplementary Figure 5E), which confirmed the conclusion that similar mechanisms appear to be involved in the development of pulmonary fibrosis in IPF and RA-ILD patients [22]. In SSc-ILD, it was also indicated that patients exhibited higher antibody levels than those without ILD [23]. The autoantibodies against the DNA-modifying enzyme topoisomerase I are frequently present in patients with diffuse cutaneous SSc and may be associated with faster progression of SSc-ILD [24]. Niklas et al. observed that in SSc and mixed connective tissue disease, lung fibrosis was positively correlated with anti-Scl-70 antibodies [25]. In terms of mechanism, research revealed that anti-MPO antibodies activate MPO, generating oxidants that drive fibroblast proliferation and extracellular matrix deposition in the distal lung parenchyma [26]. However, in our study, SSc-ILD did not show a significant increase in B cell or plasma cell populations either in the lung or peripheral blood, prompting critical evaluation of the use of B cell-targeted therapies such as rituximab in SSc-ILD [27].

In contrast to the B cell-dominant immune signatures observed in RA-ILD and IPF, our findings highlight the predominant role of T cells, particularly Th cells, in SSc-ILD. These cells were significantly elevated in the peripheral blood of SSc-ILD patients and showed a robust inverse correlation with lung function parameters. Previous studies have implicated Th cells, including Th1, Th2, and Th17 subsets, in SSc pathogenesis [28,29]. Research indicated that Th2 cells play an immunosuppressive and fibrosis-promoting role during lung inflammation and fibrosis by secreting cytokines. Th2 cells secrete IL-4, IL-5, and IL-10, which can promote the activation of B cells and induce the production of IgG1 [30]. Saito et al. demonstrated that IL-4 and IL-13 promoted fibroblast proliferation and induced fibroblast-to-myofibroblast differentiation [31]. As a new type of Th cell, Th9 was also found to promote fibroblast differentiation, activation, and collagen secretion by secreting IL-9 and promote differentiation of Th0 cells into Th2 cells by secreting IL-4 [32]. Moreover, autoreactive Th cells with a pro-inflammatory Th17 phenotype have been quantitatively associated with lung function decline in SSc patients [33]. In this study, our results support the clinical relevance of peripheral Th cell profiling as a potential biomarker for pulmonary involvement and disease progression in SSc-ILD.

Cytokines and complements play crucial roles in regulating immune responses. Th1 cells secrete TNF-α, IL-2, and IFN-γ, which are involved in the activation of macrophages and Tc cells and are associated with organ-specific autoimmune diseases. Emerging evidence suggests that Tc cells can induce apoptosis of endothelial cells, contributing to vascular injury and fibrosis [34]. Current evidence indicates that the pathogenesis of CTDs involves a complex network of various cytokines and cells, with TNF-α and IL-6 serving as key mediators in the pathogenesis of RA and SLE [35,36]. In this study, we revealed significantly elevated serum IL-6 levels in PM/DM-ILD, RA-ILD, and IPF patients compared with healthy controls. Activation of IL-6 signaling stimulated fibroblast proliferation and extracellular matrix (ECM) protein production, related to the progression of pulmonary fibrosis [37]. Additionally, *in vitro* experiments supported a potent profibrotic effect of IL-6 signaling *via* the JAK2/STAT3 and ERK pathways [38].

ILD encompasses a spectrum of pathophysiological processes, with ECM accumulation constituting a central feature of lung fibrosis [39]. ECM maintains lung homeostasis; however, the aggregation and/or degradation dysregulation of the ECM usually causes or accompanies chronic lung diseases [40]. Previous studies have shown that fibrotic lung tissue exhibits increased collagen I and α-smooth muscle actin (α-SMA) expression, contributing to increased tissue stiffness and impaired lung compliance [41]. Consistent with this, our transcriptomic analysis revealed that ECM-related pathways were highly enriched in RA-ILD, SSc-ILD, and IPF (Supplementary Figure 5C), reflecting the central role of ECM accumulation in lung fibrosis. Notably, ECM-targeted therapies, such as ECM-derived hydrogels, have demonstrated potential in reversing fibrotic phenotypes by attenuating inflammation and collagen deposition [42].

The integration of immune profiling with pulmonary function testing provides mechanistic insights into disease pathogenesis and may guide risk stratification and personalized treatment strategies in CTD-ILD. However, several limitations should be acknowledged. The retrospective, single-center design may limit the generalizability of our findings. Post-hoc pairwise comparisons were performed using Fisher’s LSD test without correction for multiple comparisons. This approach prioritizes sensitivity in detecting potential differences in this exploratory study, but increases the risk of Type I error. Therefore, the results should be interpreted with caution, and confirmatory studies with larger sample sizes and appropriate multiplicity adjustments are warranted to validate these findings. For transparency, both LSD and Bonferroni-corrected results are provided in the Supplementary Materials (Supplementary Table). Although we adjusted for disease duration, the heterogeneity of treatment regimens, as well as the lack of detailed data on dosage and treatment duration, made more refined adjustment difficult. The potential confounding effect of immunosuppressive therapy on peripheral blood immune cell profiles should be considered when interpreting these results. Additionally, longitudinal data on Th cell dynamics and their relationship with disease progression were not available, precluding assessment of their predictive value over time. Notably, after adjusting for age, sex, disease duration, smoking status, and immunosuppressive treatment, the correlations between Th cells and pulmonary function parameters in this study no longer reached statistical significance. Given the limited sample size of the SSc-ILD group, these findings warrant further validation in larger prospective cohorts. Future studies should focus on validating these findings in multicenter cohorts and exploring the differential associations of Th cell subsets, such as Th17 and Treg, with fibrosis and pulmonary function, which could refine prognostic models.

## Conclusion

This study comprehensively characterized the immune features in CTD-ILD. It established a significant correlation between peripheral immune profiles and the decline in lung function, highlighting the key role of Th cells in SSc-ILD pathogenesis. The findings suggest that elevated percentages of peripheral Th cells are strongly associated with impaired pulmonary function in SSc-ILD patients.

## Supplementary Material

Supplementary Figure.docx

Supplementary Table.xlsx

## Data Availability

The data that support the findings of this study are available from the corresponding author upon reasonable request. Direct web links to public datasets in this study are available as follows: GEO: http://www.ncbi.nlm.nih.gov/geo.
