# Supplementary material for: Connective tissue disease-associated interstitial lung disease: immune profiling and correlation of immune cells with pulmonary function in SSc-ILD
Source: Ann Med. 2026 Oct 15;58(1):2745489. doi: 10.1080/07853890.2026.2745489 (PMC13674652; doi:10.1080/07853890.2026.2745489)
Supplement: Supplementary file 1 [file IANN_A_2745489_SM5048.docx]

**Supplementary Figure List:**

1. Methods for Supplementary Figure 5

2. Supplementary Figure 1. Comparison of peripheral blood immune cell proportions after adjusting for disease duration

3. Supplementary Figure 2. Levels of cytokines in patients with CTD-ILD and IPF

4. Supplementary Figure 3. Correlation of Th cells with lung function in SSc-ILD

5. Supplementary Figure 4. Correlation of immune cells with lung function in SSc-ILD

6. Supplementary Figure 5. Distinct immune characteristics of pulmonary interstitium in CTD-ILD and IPF

**Methods of transcriptomic dataset acquisition and analysis**

Gene Expression Omnibus (GEO) is a public functional genomics data repository that provides standardized, large-scale transcriptomic datasets across diverse populations, overcoming single-center sample limitations. In this study, two publicly available RNA sequencing datasets- GSE231693 and GSE199152- were obtained. GSE231693 includes 60 lung tissue samples (20 each from IPF, SSc-ILD, and healthy controls) and was sequenced on the Illumina HiSeq 4000 platform. GSE199152 includes 27 samples (RA-ILD: n = 3; IPF: n = 20; healthy controls: n = 4) sequenced on the Illumina HiSeq 2500 platform.

Differentially expressed genes (DEGs) were identified using GEO2R with the following criteria: |log_2_ fold change| > 2 and adjusted p-value < 0.05. Gene Ontology (GO) and Kyoto Encyclopedia of Genes and Genomes (KEGG) pathway enrichment analyses are bioinformatics methods that systematically interpret gene functions and pathway associations. In this study, GO/KEGG analyses for DEGs were performed using the Metascape platform (https://metascape.org/). xCell is a computational tool designed to infer the abundance of diverse immune and stromal cell types within complex tissues using bulk gene expression data. Immune cell infiltration within lung tissues was estimated using the xCell algorithm based on bulk RNA-seq data.

**Supplementary Figure 1. Comparison of peripheral blood immune cell proportions after adjusting for disease duration**

A. Proportions of T cells, T helper (Th) cells, and cytotoxic T (Tc) cells in peripheral blood after adjusting for disease duration.
B. Proportions of B cells and natural killer (NK) cells in peripheral blood after adjusting for disease duration.
C. Lymphocyte counts in the peripheral blood of ILD patients after adjusting for disease duration. ^*^*p* < 0.05.

**Supplementary Figure 2. Levels of cytokines in patients with CTD-ILD and IPF**

A. Cytokine concentrations (IL-2, IL-4, TNF-α, IFN-γ, and IL-17) in plasma samples. **p* < 0.05, ***p* < 0.01.

**Supplementary Figure 3. Correlation of Th cells with lung function in SSc-ILD**

A. Associations between Th cell proportions and lung function in SSc-ILD. VT: Tidal Volume, BF: Breathing Frequency, MV: Minute Ventilation, IC: Inspiratory Capacity, FEV1: Forced Expiratory Volume in 1 second, FEV1% VC max: FEV1 as a Percentage of Vital Capacity, PEF: Peak Expiratory Flow, MEF75: Maximal Expiratory Flow at 75% of Vital Capacity, MEF50: Maximal Expiratory Flow at 50% of Vital Capacity, MEF25: Maximal Expiratory Flow at 25% of Vital Capacity, MEF75/25: Maximal Expiratory Flow between 75% and 25% of Vital Capacity, MVV: Maximal Voluntary Ventilation. TLC SB: Total Lung Capacity-Single Breath, RV SB: Residual Volume-Single Breath, RV% TLC SB: Residual Volume as a Percentage of Total Lung Capacity. DLCO/VA: Diffusing Capacity for Carbon Monoxide per Alveolar Volume.

B. Relationship between Th cell levels and partial pressure of oxygen (PO₂) and oxygen Saturation (SO2).

**Supplementary Figure 4. Correlation of immune cells with lung function in SSc-ILD**

A. Split-by-median analysis and continuous correlation analyses between Tc cell proportions and lung function in SSc-ILD.

B. Split-by-median analysis and continuous correlation analyses between B cell proportions and lung function in SSc-ILD.

C. Split-by-median analysis and continuous correlation analyses between NK cell proportions and lung function in SSc-ILD. **p* < 0.05, ***p* < 0.01.

**Supplementary Figure 5. Distinct immune characteristics of pulmonary interstitium in CTD-ILD and IPF**

A. Differentially expressed genes (DEGs) between ILD subtypes (SSc-ILD, RA-ILD, IPF) and normal lung tissue.
B. Overlapping DEGs of GSE231693 and GSE199152.
C. Enrichment analysis of DEGs, highlighting immune and fibrosis-related signaling pathways.
D. Infiltration of B cells and B cell plasma in lung tissues of the indicated groups.
E. Infiltration levels of T helper 2 (Th2) cells, central memory cytotoxic T cells (Tc central memory), and mast cells across groups.
